# Inhibition of Influenza A Virus Infection *In Vitro* by Peptides Designed *In Silico*

**DOI:** 10.1371/journal.pone.0076876

**Published:** 2013-10-11

**Authors:** Rogelio López-Martínez, G. Lizbeth Ramírez-Salinas, José Correa-Basurto, Blanca L. Barrón

**Affiliations:** 1 Laboratorio de Virología, Departamento de Microbiología, Escuela Nacional de Ciencias Biológicas, Instituto Politécnico Nacional, Carpio y Plan de Ayala S/N, Casco de Santo Tomás, México D.F., México; 2 Laboratorio de Modelado Molecular y Bioinformática, Escuela Superior de Medicina, Instituto Politécnico Nacional, Plan de San Luis y Díaz Mirón S/N, Delegación Miguel Hidalgo, México D.F., México; University of Edinburgh, United Kingdom

## Abstract

Influenza A viruses are enveloped, segmented negative single-stranded RNA viruses, capable of causing severe human respiratory infections. Currently, only two types of drugs are used to treat influenza A infections, the M2 H^+^ ion channel blockers (amantadine and rimantadine) and the neuraminidase inhibitors (NAI) (oseltamivir and zanamivir). Moreover, the emergence of drug-resistant influenza A virus strains has emphasized the need to develop new antiviral agents to complement or replace the existing drugs. Influenza A virus has on the surface a glycoprotein named hemagglutinin (HA) which due to its important role in the initial stage of infection: receptor binding and fusion activities of viral and endosomal membranes, is a potential target for new antiviral drugs. In this work we designed nine peptides using several bioinformatics tools. These peptides were derived from the HA1 and HA2 subunits of influenza A HA with the aim to inhibit influenza A virus infection. The peptides were synthetized and their antiviral activity was tested *in vitro* against several influenza A viral strains: Puerto Rico/916/34 (H1N1), (H1N1)pdm09, **s**wine (H1N1) and avian (H5N2). We found these peptides were able to inhibit the influenza A viral strains tested, without showing any cytotoxic effect. By docking studies we found evidence that all the peptides were capable to bind to the viral HA, principally to important regions on the viral HA stalk, thus could prevent the HA conformational changes required to carry out its membranes fusion activity.

## Introduction

Influenza A viruses are enveloped, segmented negative single-stranded RNA viruses, belonging to the *Orthomyxoviridae* family, capable of causing several respiratory diseases in humans, varying from upper acute respiratory infections to severe diseases like pneumonia [1]. Influenza A virus infection initiates with the attachment of the viral surface glycoprotein hemagglutinin (HA) to sialic acid receptors located on the cell surface. The HA encoded by the viral genome segment 4, is classified as a surface glycoprotein type I, is the main viral antigenic determinant, and has been typed in 20 subtypes (H1-H20) [2,3]. The HA is synthesized as a polypeptide HA0 precursor, which contains a signal sequence, a proteolytic cleavage site, a hydrophobic sequence known as the fusion peptide, and a transmembrane anchor domain in the carboxy-terminal end, followed by a cytoplasmic tail. The HA0 polypeptide is folded and assembled in homotrimers in the endoplasmic reticulum (ER) [2]. A posttranslational proteolytic cleavage generates the HA1 and HA2 subunits which are covalently linked by a disulfide bond [2,3,4]. The HA homotrimer forms a large membrane-distal globular domain and an elongated membrane-proximal domain (stalk region). The distal domain is made only by the HA1 polypeptide and contains the sialic acid-receptor binding site (RBS) and a vestigial esterase (VES) subdomain [5]. The stalk region is mainly formed by the HA2 polypeptide, which contains the Fusion subdomain (F) and the N- and C- terminal segments of the HA1 polypeptide (F´ fusion subdomain) [6,7].

Once Influenza A virus HA has bound to the cellular receptor, the viral particle is internalized into the endocytic compartment [8] where low-pH induces several structural changes on the HA, making a loop-to-helix transition of an inter-helical loop (B loop). This loop-to-helix transition enables extension of the central coiled-coil and facilitates relocation of the fusion peptide toward the target membrane [9,10,11,12,13,14,15]. Currently, two types of antiviral drugs are available against influenza A virus infections, the neuraminidase inhibitors (NAI) that block the viral progeny release from the infected cell. The other group of compounds is the amantadanes or blockers of the M2 viral ion channel, which prevent viral uncoating process [16]. Nowadays, two NAI are available, Olsetamivir and Zanamivir, however resistance has been reported for A/H5N1, A/H3N2, and A/H1N1 seasonal and pandemic strains [17,18,19]. The amantadanes, like amantadine and rimantadine are not recommended due to their secondary effects and high level of resistant strains, above 98% [20,21]. 

To search for new antiviral drugs, several strategies have been used, one approach has been the screening of thousand compounds against influenza A virus; and other is based on the design of antivirals against specific an important viral molecules [22]. The design of antiviral peptides (AVPs) has been considered an important strategy to control viral infections either by blocking viral attachment or entry into host cells or by using peptides coupled to other molecules to penetrate into the cell and then, interfere with internal fusion or replication events [22,23,24]. Several antiviral peptides against different viruses have been found by using the phage displayed strategy, such as a murine brain cDNA phage display library against West Nile Virus, or a disulfide constrained heptapeptide phage display library against avian influenza virus H9N2 [25,26,27].

It is known that bioinformatics and molecular modeling tools are a very useful approach to design new antiviral drugs [28,29,30]. Those computer-based methods allow designing peptides with high-affinity for a specific target protein, such as essential proteins involved in the initial steps of the viral infectious cycle. By using this approach, peptides derived from Dengue virus E protein have been designed to inhibit viral binding or the fusion event to the host cell [28,31]. For influenza A virus two proteins, HB36 and HB80, were designed to bind a conserved surface patch on the stalk of the influenza hemagglutinin (HA) of the 1918 H1N1 pandemic virus and it has been suggested that both proteins could be useful for therapeutics or diagnosis procedures [29]. 

Our group developed an algorithm to design several peptides to inhibit influenza A viral infections *in vitro*. These antiviral peptides (AVPs) were derived from the influenza A virus HA, specifically from highly conserved regions [30]. In this study, we present the *in vitro* antiviral evaluation of the designed AVPs against influenza A virus using several influenza A strains and also, docking studies to elucidate the probable antiviral mechanism. 

## Material and Methods

### Cells and viruses

MDCK cells were grown and maintained in DMEM F:12 medium supplemented with 10% of fetal bovine serum. Each influenza A virus strain: avian H5N2, swine classic H1N1, Puerto Rico/916/34 (H1N1), and pandemic (H1N1)pdm09, were propagated in MDCK cells using DMEM F:12 medium supplemented with bovine serum albumin 1 %, HEPES 20 mM, and 4.5 µg/ml of trypsin. Cells were incubated at 37°C for 3-6 days, until cytopathic effect (CPE) was observed. Supernatants were clarified by centrifugation at 800 xg for 5 min, and aliquots were stored at -70°C before use. Virus titer was determined by the TCID_50_ method.

### Design and analyses *in silico* of antiviral peptides

The design of potentially antiviral peptides (AVPs) against influenza A virus was carried out as previously described [30]. Briefly, the HA1 and HA2 sequences available until August 2011 were separately downloaded from the NCBI database (National Center for Biotechnology Information) [http://www.ncbi.nlm.nih.gov/genomes/FLU/Database/]. The sequences were clustered according to the subtype of HA and each cluster was aligned with ClustalX2 [32] to obtain a consensus sequence for each HA subtype. A second alignment using TCOFFEE [http://www.ebi.ac.uk/t-coffee/] [33,34] was carried out with all the consensus sequences previously obtained, to detect regions with highly conserved amino acids sequence to generate a global consensus sequence, which was edited with GeneDoc v.2.7.000, BioEdit v.7.0.9.0 and the WEBLOGO server [http://weblogo.berkeley.edu.org/] [35]. The highly conserved regions from HA1 and HA2 subunits represented in the consensus sequences were separately analyzed using several bioinformatics tools to determine physicochemical properties such as, hydrophobicity and flexibility, using the scales Kyte-Doolittle and Average Flexibility Index, respectively [http://web.expasy.org/protscale/] [36,37]. Antigenicity was predicted using the Parker’ scale with the Hydrophilicity Prediction program [http://tools.immuneepitope.org/tools/bcell/iedb_input] [38,39]. Chemical charge was determined with the EMBOSS server [40,41]. Finally, the highly conserved regions (>80% conserved amino acid sequence) with a size of 12 to 20 amino acids length, which at the same time showed to be hydrophilic, flexible, exposed and with chemical overall charge were chosen as probably AVPs. 

### Docking studies

Protein-protein docking was done by ClusPro.2.0 server [http://cluspro.bu.edu/login] [42], which is a fully automated web-based program based on CAPRI (critical assessment of prediction of interactions), using the HA (H1N1)pdm09 3D structure [PDB:3LZG] as a target for the AVPs 3D structures. The AVPs 3D structures were modeled using the PEP-FOLD program [http://bioserv.rpbs.univ-paris-diderot.fr/PEP-FOLD/] [43,44]. The best 3D model for each AVP was selected according to PEP-FOLD server, considering the lowest energy model (sOPEP, score Optimized Potential for Efficient structure Prediction) that indicates peptide stability [44]; and the highest tm value (template modeling score) that indicates the quality of peptide structure according the template alignments used [45]. The complexes were analyzed using Chimera v1.6rc and the server CMA (Contact Map Analysis) [ligin.weizmann.ac.il/cma/]) [46]. Docking results were visualized using Chimera v1.6rc and PyMOL 1.3 program. We selected the models with the lowest balanced coefficients of free energy values, ΔG (kcal/mol), which meant they were energetically favorable; and also, the lowest cluster complex size, which is related to a high frequency of interactions between receptor (HA) and ligand (AVP) at the same site.

### Peptides synthesis and cytotoxicity assays

The AVPs were synthesized at Invitrogen^TM^ USA with minimum of 95% of purity. The AVP cytotoxicity assays were carried out in MDCK cells using the MTT reduction method [47]. Briefly, MDCK cells were seeded at 2X10^4^ cells/well in 96 microwell plates and incubated at 37°C with 5% CO_2,_ 24 h before use. Each AVP was separately diluted to obtain different concentrations, and then, a final volume of 100 μl of each concentration, was added to MDCK cells. After 24 h incubation period at 37 °C in a humidified 5% CO_2_, 10 μl of MTT reagent (5 mg/ml, 3-(4,5-dimethyl-2-thiazolyl)-2,5-diphenyl-2H-tetrazoliumbromid in PBS) were added to each well. After 4 h of incubation, the MTT-reagent was removed and the formazan generated in the cells was dissolved with 50 μl of 15% w/v sodium dodecyl sulfate in 0.02 M HCl. The absorbance of each well was measured at 570 nm [48]. Untreated control cells were included. The 50% cytotoxic concentration (CC_50_) was defined as the peptide concentration that reduced cell viability by 50%, compared to untreated controls, and it was calculated as [(A-B)/A]X100, where A and B are the OD_540_ of untreated and treated cells, respectively [49,50,51]. 

### Antiviral assays

The antiviral assays were done in a similar way as it was described for the cytotoxicity assay; but in these cases, virus and peptides were added simultaneously to the cells [52]. Each influenza A virus was used at 100 TCID_50_/well. The mixture cells-virus-AVP was incubated at 37°C in atmosphere of 5% CO_2,_ until the viral control showed >80% CPE. Viral infection was detected by the MTT method as describe above. The percent protection was calculated as [(A-B)/C-B)]x100, where A, B and C corresponded to the absorbance of treated infected, untreated infected, and untreated uninfected cells, respectively. The 50% viral inhibitory concentration (IC_50_) for each AVP was determined as the peptide concentration that achieves 50% protection of treated infected cells [49,50,51]. 

## Results and Discussion

### Design and analyses *in silico* of antiviral peptides

**Figure 1:**
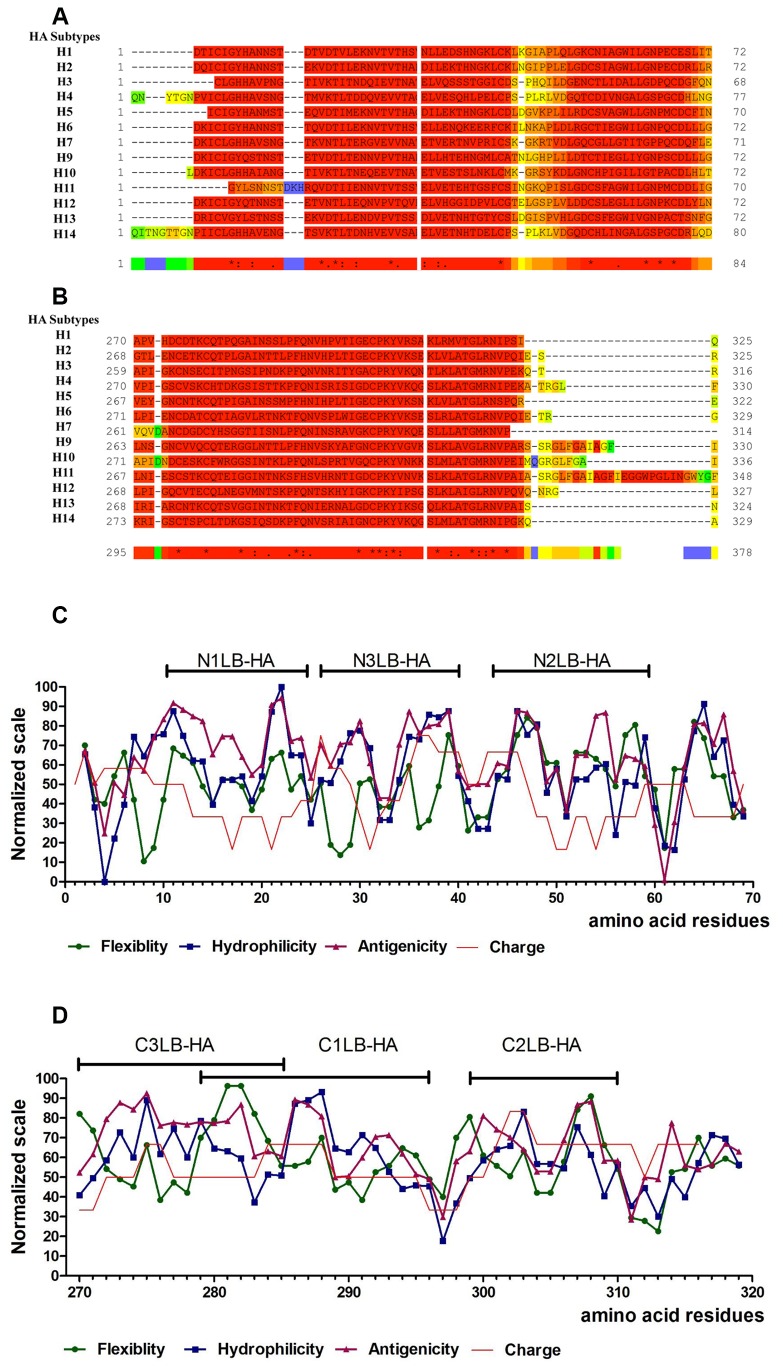
Conserved amino acid sequences located at the N-t and C-t ends of HA1 subunit and physicochemical parameters used to design the AVPs. **1a**) **and 1B)** T-Coffee alignment of thirteen consensus sequences obtained for the H1, H2, H3, H4, H5, H6, H7, H9, H10, H11, H12, H13, and H14, HA subtypes. A) Alignment of the N-t end of the HA1 subunit; B) Alignment of the C-t end of the HA1 subunit. Color´ scale: blue-bad to red-good. This color scheme is named the CORE index and is a mean of assessing the local reliability of a multiple sequence alignment. 1C) and 1D) Normalized physicochemical properties determined by *in* *silico* analysis of the N-t and C-t ends of the HA1 subunits. The lines above indicate the regions selected to design the AVPs, and the name of the AVP.

A total of 5,918 HA1 subunit sequences belonging to 13 of 16 HA subtypes were analyzed. After the alignment and edition process, a 93 TCOFFEE score was obtained. For the subunit HA2 alignment we used the complete amino acid sequence (560-570 amino acid residues) of each of the 16 reference HA subtypes, and a 95 score was obtained with T-COFFEE express. Both, HA1 and HA2 subunits showed a highly conserved amino acid sequence of approximately 50 and 40 amino acids length, located at the N-t and C-t end of the HA1 subunit, respectively (Figures 1A, 1B); and 80 amino acids length at the N-t of the HA2 subunit (data not shown). 

Once we detected these conserved regions in the HA subunits, they were submit analyzed for several physicochemical parameters to design the antiviral peptides (AVPs). Since it is known that due to the nature of the membrane fusion process that allows viral entry into the cell, functional regions of the viral glycoproteins involved in this event, need to be accessible (surface exposed), hydrophilic, and flexible. These properties have been found to be important for the protein-protein interactions, necessary for the activation of the fusion event [53]. Therefore, regions of the glycoproteins with these physiochemical properties have been used for designing antiviral peptides [53,30]. 

The physicochemical *in silico* analysis of the conserved regions of the HA showed several short regions in both, N-t and C-t ends of the HA1 subunit, which were used to generate the AVPs, due that these regions presented the properties that we were looking for: hydrophilic, flexible, exposed (antigenic) and with chemical overall charge (Figures 1C, 1D). The AVPs were derived from the amino acid residues located at the positions 10-24, 44-59 and 26-40 of the N-t end; and 279-296, 299-310, 270-285 of the C-t end. Both regions corresponded to the F’ subdomain of 3D HA structure [8]. The AVPs were labeled as N1LB-HA, N2LB-HA, N3LB-HA, and C1LB-HA, C2LB-HA, C3LB-HA, for N-t and C-t end, respectively. The AVPs from the HA2 subunit were derived from the amino acid residues located at the positions 410-421, 489-510, and 517-537. These AVPs were labeled as PHGB-1, PHG-3 and PHGB-4, and corresponded to the HA stalk region [54].

### AVPs cytotoxicity

Cytotoxicity assays carried out with the HPLC-purified peptides synthesized by Invitrogen® did not show any change in cell morphology, and the MTT reduction assay confirmed the low values of toxicity. The maximum toxicity level was 15% and was detected only at the highest concentration of the AVPs, 2.5 mM (data not shown), result that is in agreement with other previous reports [22,26]. 

### Antiviral effectiveness of the designed AVPs

**Figure 2:**
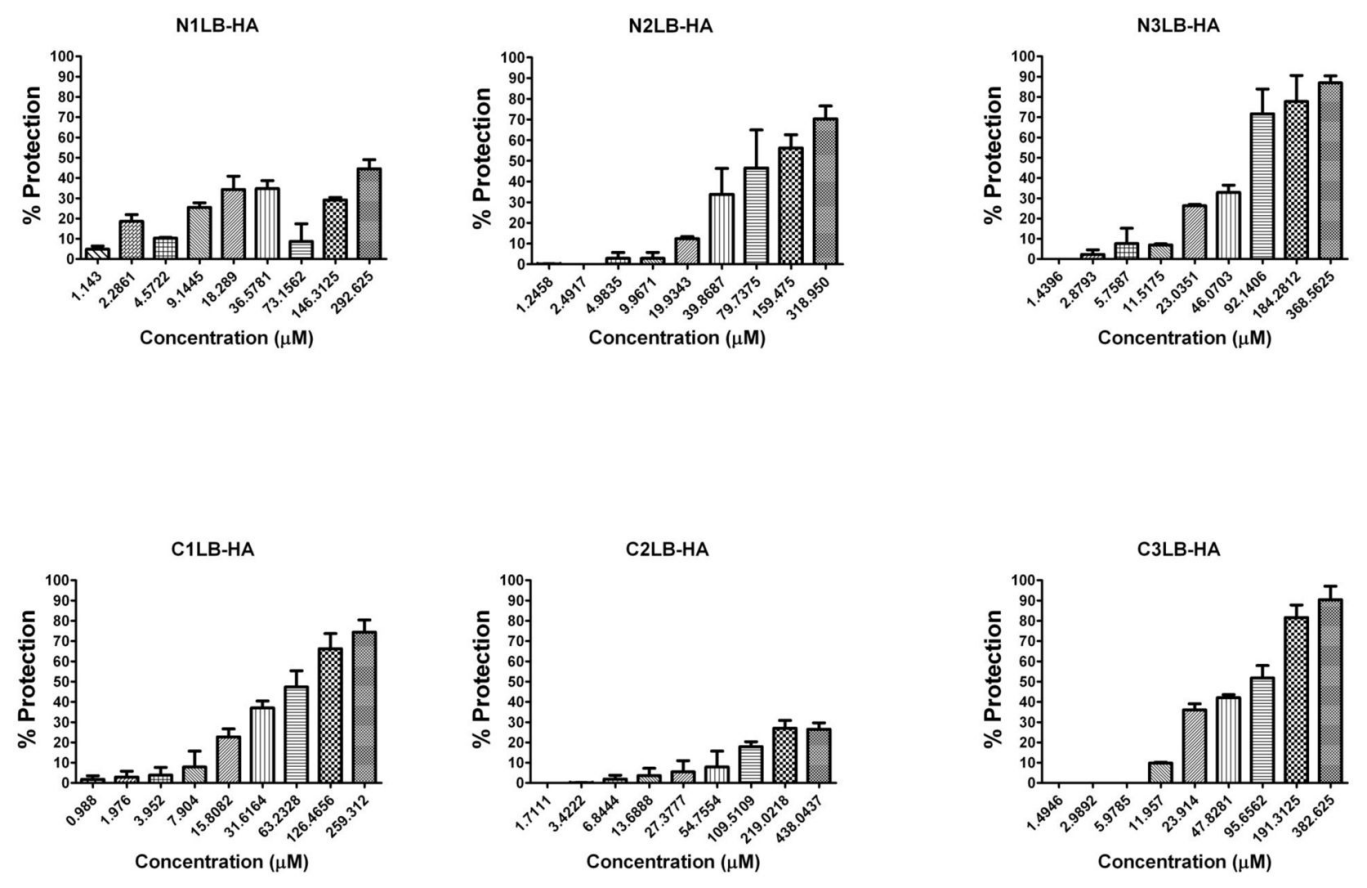
Antiviral activity against influenza A Puerto Rico/916/34 (H1N1) viral strain by AVPs derived from HA2 subunit. It is expressed as % of cell protection against viral infection, measured by MTT assay. The upper line in each figure indicates the tested AVP.

**Figure 3:**
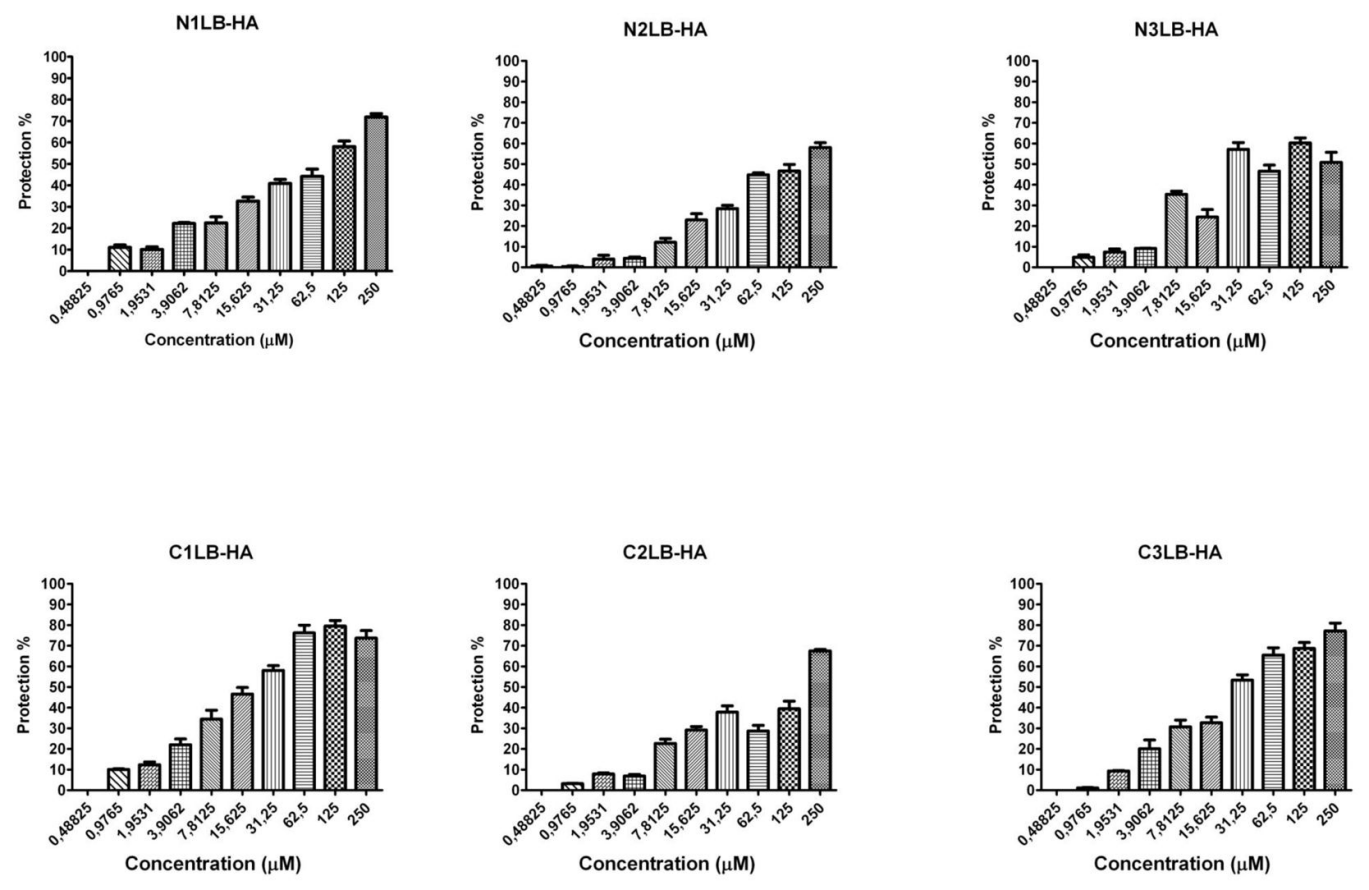
Antiviral activity against influenza A (H1N1)pdm09 viral strain by AVPs derived from HA1 subunit. It is expressed as % of cell protection against viral infection, measured by MTT assay. The upper line in each figure indicates the tested AVP.

**Figure 4:**
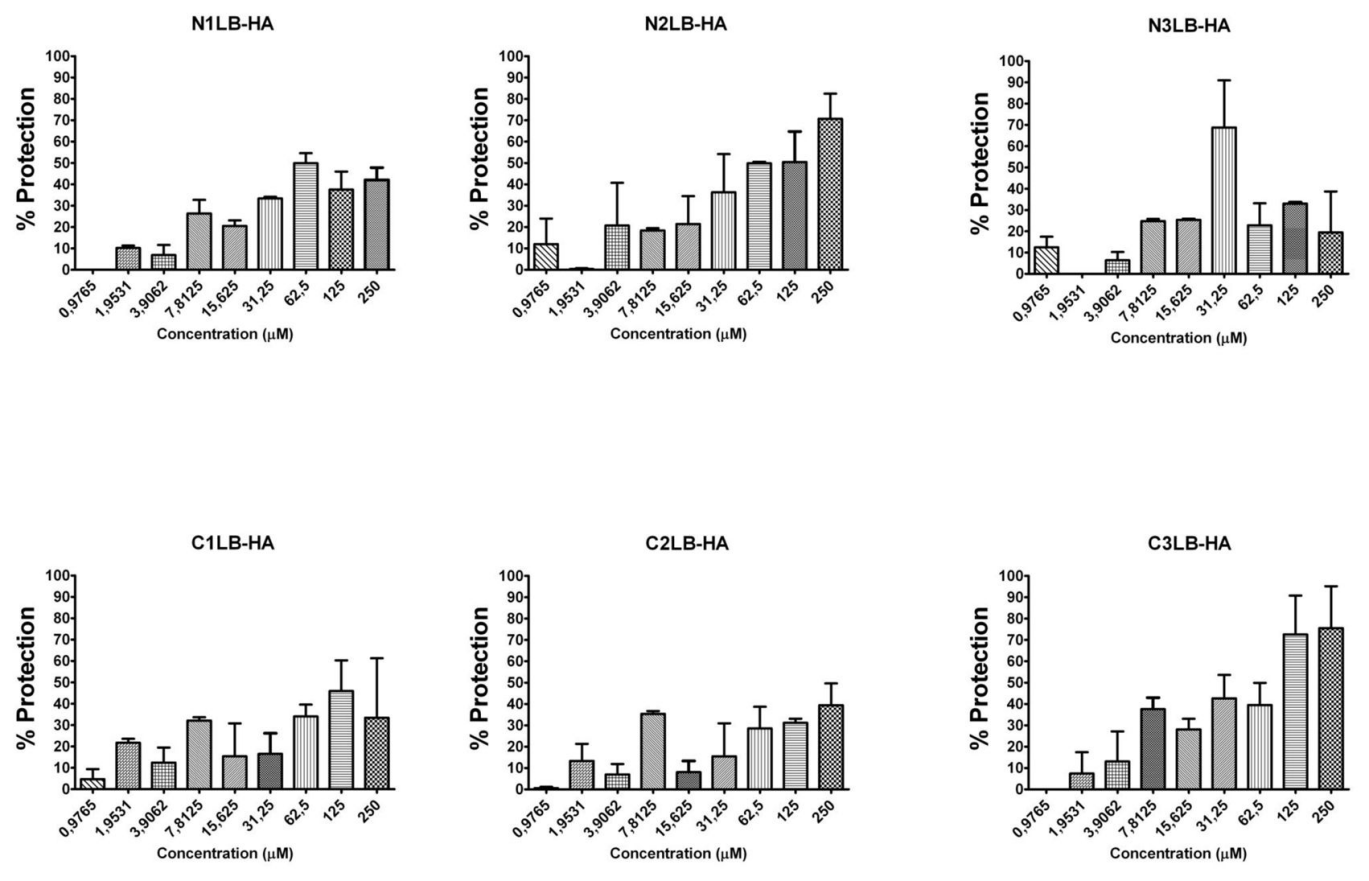
Antiviral activity against influenza A H1N1 swine viral strain by AVPs derived from HA1 subunit. It is expressed as % of cell protection against viral infection, measured by MTT assay. The upper line in each figure indicates the tested AVP.

**Figure 5:**
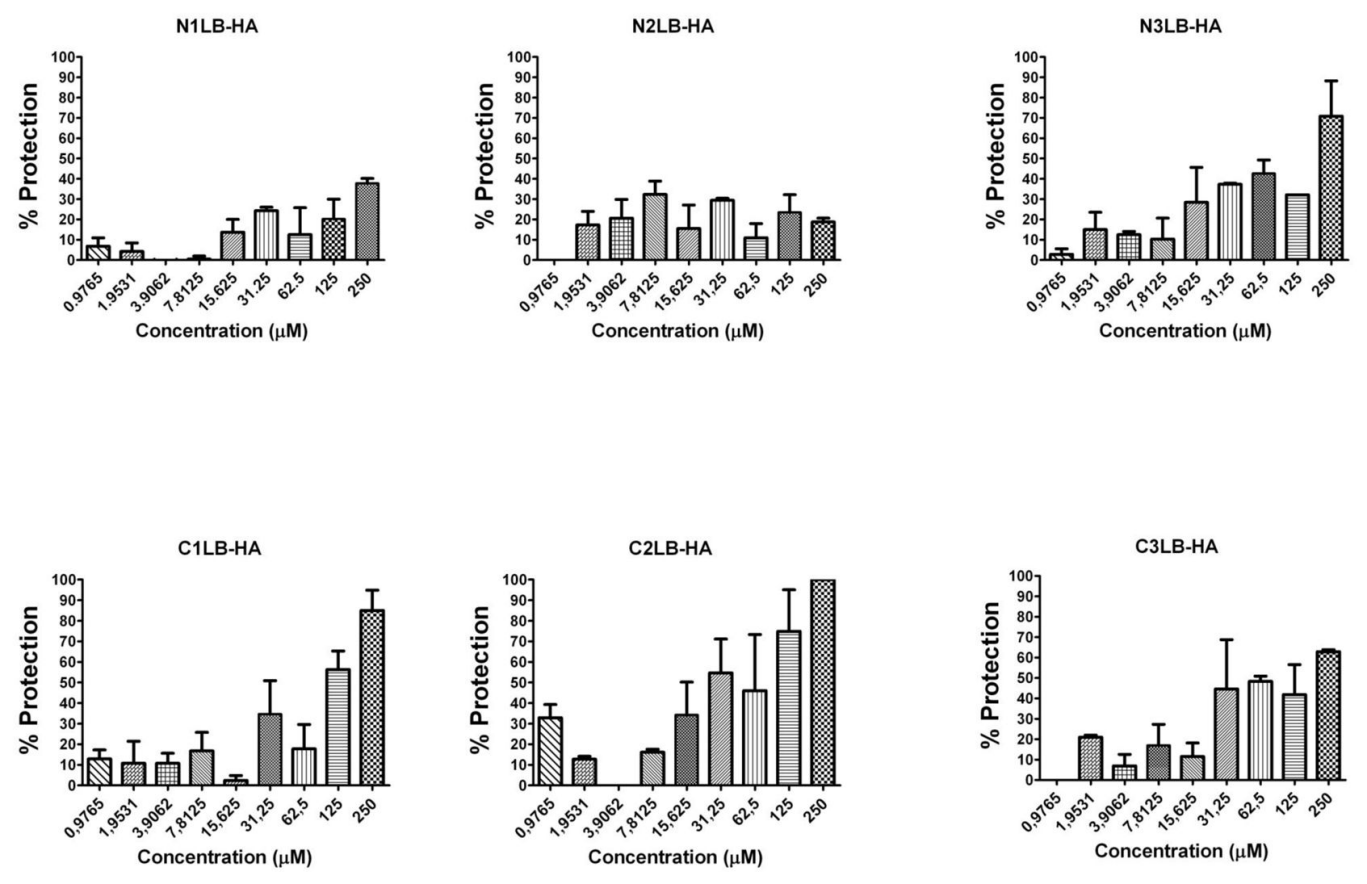
Antiviral activity against influenza A H5N2 avian viral strain by AVPs derived from HA1 subunit. It is expressed as % of cell protection against viral infection, measured by MTT assay. The upper line in each figure indicates the tested AVP.

**Figure 6:**
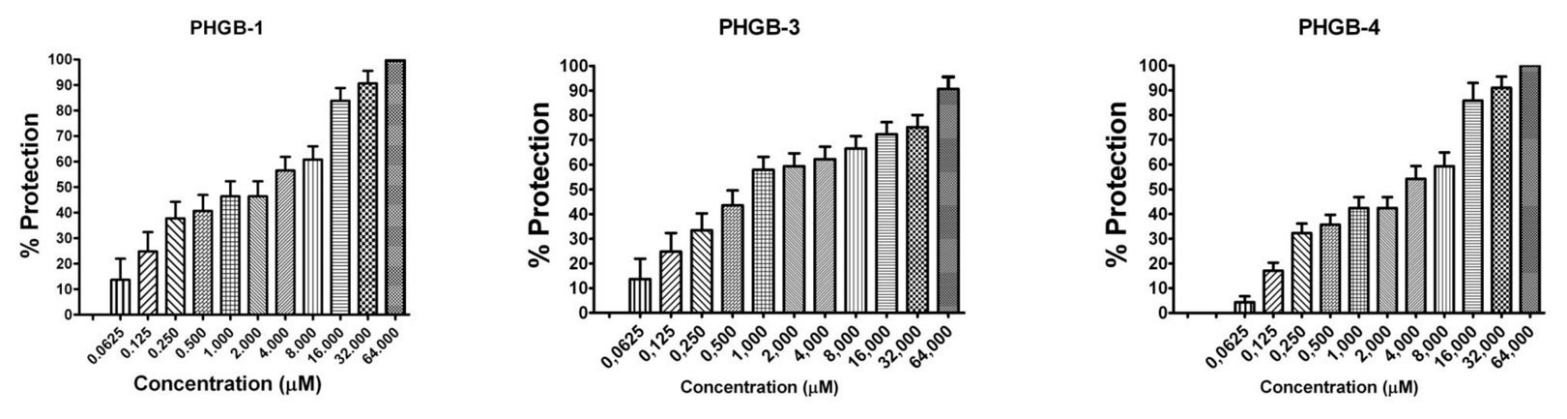
Antiviral activity against influenza A Puerto Rico/916/34 (H1N1) viral strain by AVPs derived from HA2 subunit. It is expressed as % of cell protection against viral infection, measured by MTT assay. The upper line in each figure indicates the tested AVP.

The antiviral assays of the designed AVPs showed that all of them were capable to inhibit the four influenza A viral strains, since after 96 h of being the virus in presence of each of the AVPs, most of the cells did not show evidence of CPE, compared to the untreated infected cells, which showed 100% of CPE. The CPE was characterized by cellular detachment, as well cell rounding swelling and finally cellular death. Even more, CPE inhibition was confirmed by the MTT assay, and the protection of the cells against viral infection was directly related to the AVP concentration as a dose-response effect (Figures 2-6). These results confirmed that peptides derived from the protein-protein interface could block the viral infection probably due that they mimic the modes of binding of its original domain to its specific partner protein [55]

The effectiveness of each AVP was compared by calculating its lowest concentration capable to inhibit 50% of the viral infection (IC_50_) (Table 1). All the AVPs derived from the HA1 subunit were effective against the four influenza A viral strains, and most of them required a concentration within a range of 20 to 74 µM to obtain one IC_50_. Data that is within the range of other antiviral peptides, such as a cyclic and a linear peptide (100 µM) tested against avian influenza H9N2 [26,27] or the new chemical fusion inhibitors [56], or even more, the zanamivir antiviral assays against several influenza A virus strains in MDCK cells has reported IC_50_ from 4 to 58.3 µM; although the zanamivir IC_50_ determined by the enzymatic inhibition method of the viral neuraminidase activity has been reported in range of 2.2 to 30 nM [57]. 

**Table 1:** Comparison of the 50% inhibitory concentration (IC_50_) required by each AVP to inhibit influenza A viruses.

**Viral inhibitory concentration IC_50_ (µM)**				
**AVP**	Influenza A strain			
	PR/916/34 (H1N1)	Pandemic (H1N1)pdm2009	Swine classic (H1N1)	Avian (H5N2)
**N1LB-HA**	≥250	33.97	50.83	≥250
**N2LB-HA**	27.01	30.46	30	≥250
**N3LB-HA**	20.46	40.23	20.19	35.45
**C1LB-HA**	25.43	27.94	62.16	22.41
**C2LB-HA**	≥250	74.07	≥250	27.89
**C3LB-HA**	27.21	26.45	28.3	30.95
**PHGB-1**	32	ND	- ND	-ND
**PHGB-3**	33	ND	- ND	- ND
**PHGB-4**	33	ND	- ND	- ND

ND=No determined

The AVPs derived from the N-t end inhibited the three H1N1 influenza viruses at an IC_50_ 20-50 µM. In contrast the avian H5N2 strain required a higher IC_50_ (≥250 µM). The AVPs derived from the C-t end were highly effective against the avian H5N2 viral strain (22-31 µM), but also against the H1N1 strains, except the C2LB-HA peptide. For the three AVPs derived from the HA2 subunit, we found similar IC_50_ 32-33 µM to inhibit the influenza A PR/916/34 (H1N1) viral strain (Table 1). 

All these antiviral assays demonstrated that the designed AVPs were effective to inhibit different influenza A virus strains of human, swine and avian origin, even that the avian strain had a different HA subtype compared to the other strains. And furthermore, it is known that the phylogenetic tree of influenza A virus based on HA subtype H1N1 can be separated in three phylogenetic subtypes according to the host: avian, swine or human [58].

Our strategy showed that the designed AVPs had an inhibitory activity against influenza A virus either with HA subtype H1 or H5. But, because our AVPs were derived from highly conserved amino acid sequences of the HA stalk region, they probably could be effective against any HA subtype. Since it was demonstrated that the HA stalk contains highly conserved regions among all 16 influenza A HA subtypes [59,60,61]. But it would be necessary further assays to confirm our proposal as well as to find out the mechanism for the antiviral activity. 

We suggested as probable mechanism for the antiviral activity of the designed AVPs, that each AVP could bind to the HA, blocking its conformational changes inside the endosome, preventing the fusion event [62], due that protein-protein interactions are necessary to achieve the conformational changes required to activate the fusion mechanism [53,30]. In order to analyze this proposal we carried out the protein-protein docking studies. 

### Docking studies

**Figure 7:**
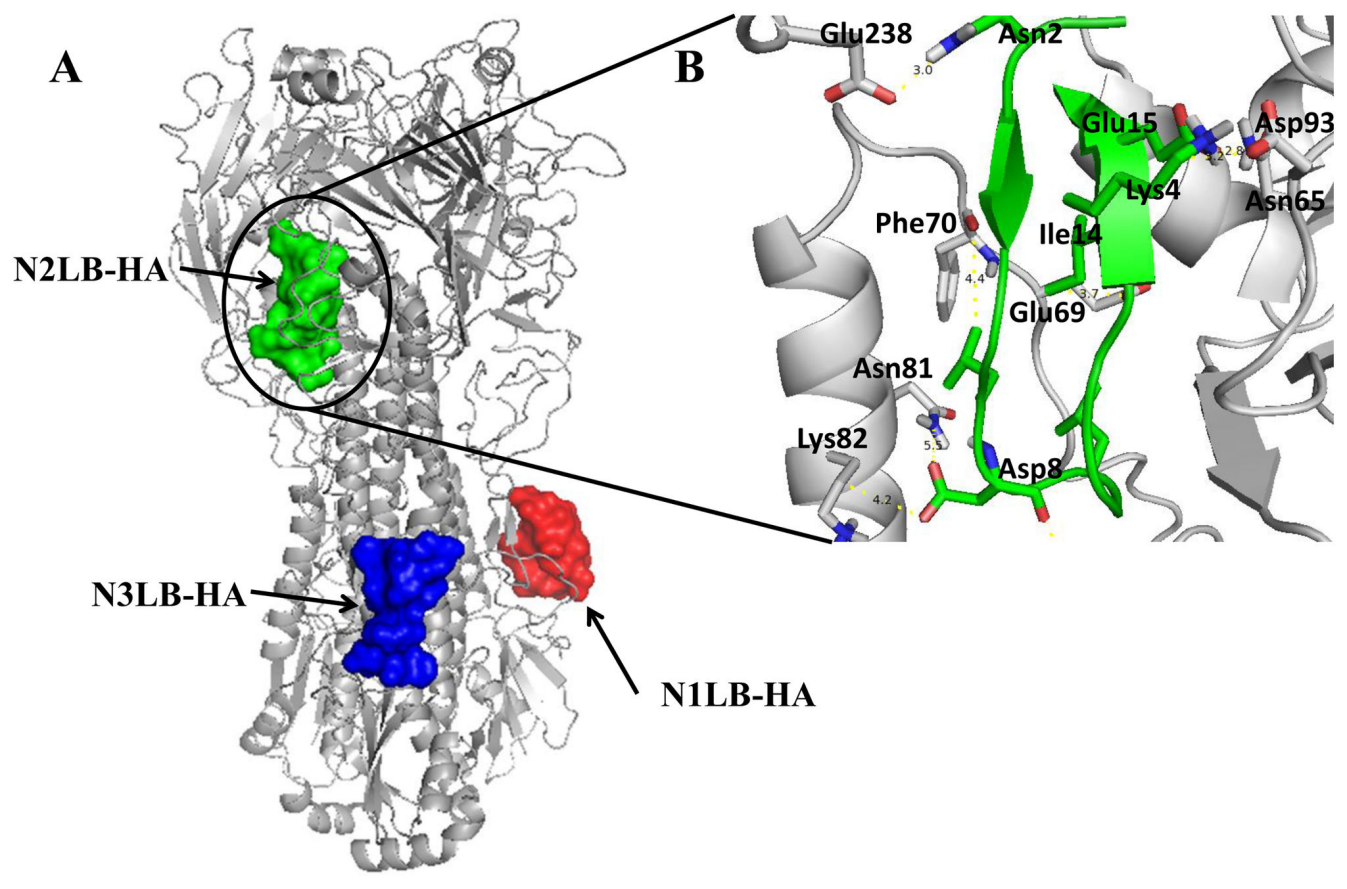
Interaction sites among AVPs derived of N-t of the HA1 subunit targeting influenza A HA (3LZG). A) Sites of interaction. B) An amplification of **N2LB-HA** interaction with the distal domain of HA.

Due that *in vitro* antiviral activity assays (Figures 2-6 and Table 1) showed that the three AVP derived from the N-t end HA1 were mainly effective against the H1N1 viral strains (IC_50_ 20-50 µM), a docking analysis was performed with each AVP 3D (N1LB-HA, N2LB-HA and N3LB-HA) model against the trimeric structure of (H1N1) HA protein (PDB:3LZG). Docking results showed multiple hydrogen bonds and electrostatic interactions between each AVP and the HA. These interactions were located at important HA domains, such as the F´, Helix A, Loop B subdomains in the stalk region; but also, the RBS and VES in distal HA region [30] (Figure 7A). Furthermore, these AVPs were also capable to bind to several *in silico* predicted linear and discontinuous epitopes located on the HA stalk and globular regions (Table 2). All these bindings could interfere with the conformational changes required by the HA to expose its fusion subdomain which carries out the fusion activity [5,6,7]. 

**Table 2:** Interactions among influenza A HA and AVPs derived from the subunits HA1 and HA2*.

**AVP (size)**	**Docked energy**	**Predicted HA Epitope(s) Interacting with the AVPs**	
		Linear^a^/(HA subdomain)	Discontinous^b^ HA subdomain/# of residues interacting
**N1LB-HA (15-mer)**	-499.3	IEGGWTGM**VDGW**/(F) DTLCIGY**H**A**NNSTD**/(F´)	F’, Fusion peptide/11
**N2LB-HA (16-mer)**	-649.3	**E**PGDKITFEATGNLVVPRYAFA/(RBS) **TSSD**N**GT**CYP/(VES) EIAIRPKV**R**DQEG/(RBS) **S**DTPVHDCN/(RBS, F´)	RBS, VES/10
**N3LB-HA (15-mer)**	-673.0	IEGGWTG**MVDGW**/(F) DTLCIGY**H**A**N**NSTD/(F´)	F’, Fusion peptide, Helix A/8
**C1LB-HA (18-mer)**	-704.0	G**N**P**E**CESLSTAS/(VES) **TSSDNGTC**Y**P**/(VES) EIAIRPK**VR**DQEG/(RBS) **S**DTPVHDCN/(RBS, F´)	VES, RBS/14
**C2LB-HA (12-mer)**	-646.0	none	none
**C3LB-HA (16-mer)**	-532.6	DTLCIGY**H**A**N**NSTD/(F´) IEGGWTGM**VDGW/**(F) GYHHQNEQGSGYA**A**D**L**K/(Helix A)	F’, Fusion peptide, Helix A/8
**PHGB-1 (12-mer)**	-605.7	H**H**PSTSADQQSLYQNADTY/(RBS) **E**IAI**R**PKVRDQEG/(RBS)	RBS/2
**PHGB-3 (22-mer)**	-921.7	**TSSD**N**GT**CY**P**/(VES) S**D**TPVHDCN/(RBS, F´) **EPG**DKITFEATGNLVVPRYAFA/(RBS)	VES, RBS/14
**PHGB-4 (21-mer)**	-766.2	**E**PGDKITFEATGNLVVPRYAFA/(RBS)	RSB/1

* Target influenza A HA (3LZG). The epitopes were predicted *in silico* by using http://tools.immuneepitope.org/tools/ElliPro/iedb_input [68]

^a^ In bold are indicated the HA amino acid residues located within the predicted linear epitope(s) interacting with the AVP.

^b^ Indicate the HA subdomains whose amino acid residues are located within predicted discontinuous epitope(s) interacting with the AVP.

To analyze in more detail the AVP interactions to HA, we chose the N2LB-HA AVP [30], (Figure 7B, Table 2). Many interactions were found with several HA subdomains, such as: in the **VES**, Asn^65^-Glu^15^, Glu^89^-Ser^13^, Pro^90^-Asp^11^, Ser^92^-Glu^15^, Asp^93^-Lys^4^, Thr^96^-Leu^1^, Gly^100^-Leu^1^, Phe^102^-Leu^1^, Try^105^-Glu^15^, Glu^106^-Ile^14^, and Arg^109^-Ile^14^; in the **RBS** Ser^207^-Asn^2^, Arg^208^-Asn^2^, Glu^238^-Asn^2^, Ile^269^-Cys^12^, Pro^284^-Leu^9^, and His^298^-Asp^8^; in the F´, Ile^300^-Leu^9^; in the **Loop B**, Gly^67^-Leu^9^, Lys^68^-Leu^7^, Glu^69^-Ile^14^, Asn^71^-Gly^16^, and His^72^-Gly^16^. But besides that, interestingly, we found the HA Phe^70^ interacted with Ile^14^-AVP, and this interaction could be very relevant, since it is known, that although low pH is the sole trigger for fusion in influenza virus HA, it does this in a well-regulated, stepwise manner. At neutral-pH the B loop displays a “collapsed” conformation and is closely packed against the central coiled coil. The interactions are mostly hydrophobic and involve two highly conserved phenyl alanine residues (Phe^63^ and Phe^70^). Both residues are considered a conformational lock for HA loop B, and at low pH they are released from their binding socket, destabilizing the loop B conformation to form a triple-stranded post-fusion structure [9]. Therefore, if the N2LB-HA peptide is bound to HA Phe^70^could maintain the lock for the loop B, even at acidic pH (Figure 7B), and this this interaction could block the viral infection due that the fusion peptide is unable to be exposed. Besides that, the side-chain of Asp^8^ from N2LB-HA interacted also with HA side-chain of Asn^81^ and Lys^82^ making hydrogen bond and electrostatic interactions, respectively, in the Helix B subdomain (within the hepta-repeat2) (Figure 7B), thus interaction could also help to block the exposure of the fusion peptide. Therefore these docking results are in agreement with the antiviral activity observed against the H1N1 viral strains.

**Figure 8:**
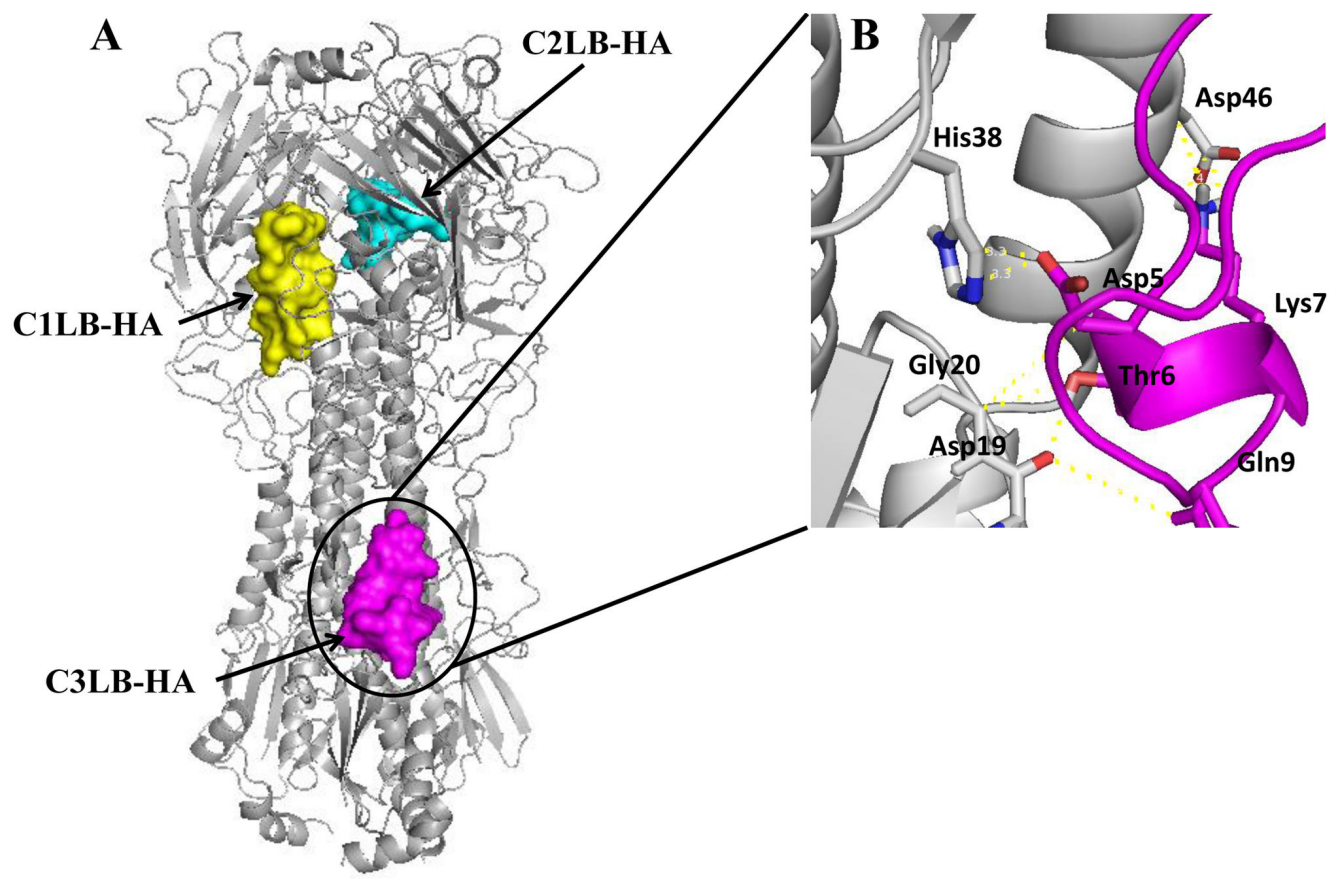
Interaction sites among AVPs derived of C-t of the HA1 subunit targeting influenza A HA (3LZG). A) Sites of interaction. B) An amplification of C3**LB-HA** interaction with the proximal domain of HA.

Due that the C3LB-HA AVP was highly effective against the H1N1 and H5N2 strains (IC_50_ 26-31 µM) (Table 1, Figures 2-6), we analyzed in more detail its interactions with the HA (Figure 8B). Docking with C3LB-HA indicated that binding could occur in the HA membrane proximal domain, specifically by interaction with the Helix A, fusion peptide, and F’ subdomains, through 101 contacts [30]. We found several interactions involving side-chains of amino acid residues from HA Helix A and F´ subdomains: with the Helix **A**: Leu^38^-Thr^6^, Thr^41^-Thr^6^, Gln^42^-Lys^7^, Ile^45^-Lys^7^, Asp^46^-Lys^7^, Thr^49^-Asn^3^, Val^52^-Ile^1^, Asn^53^-Ile^1^ and, Ile^56^-Ile^1^. With the **Subdomain F’** the interactions were: His^18^-Pro^11^, His ^38^-Asn^3^, Val ^40^-Gly^2^, Ser^292^-Ile^1^, Leu^292^-Ile^1^, and Thr^318^-Asn^3^. Side-chain of amino acid residues from HA fusion peptide Val^18^, Asp^19^, Gly^20^ and, Trp^21^ were in contact with Asn^3^, Asp^5^, Thr^6^, Gln^9^ and Pro^11^ residues of C3LB-HA AVP, making principally hydrogen bond interactions. Interestingly we found that the interaction between Gly^20^ and both Thr^6^ and Asp^5^ was stabilized by the others amino acids side-chains surrounding (Figure 8B), and all of them could be helping to keep the HA hairpin structure closed, not allowing the two conformational changes required to release and anchor the fusion peptide into the endosome membrane [63]. Moreover, it is known that HA Asp^19^ has to be neutralized by the endosome acid pH in order to participate in the opening of the fusion pore, but due to its interaction with the AVP Thr^6^ and Gln^9^, its function could be blocked, resulting in an inhibition of the viral infection [64]. Due that it has been demonstrated by using antibodies such as C179, 12D1, FI6V3, and CR8020 which interact with the stalk region, that the Helix A subdomain is highly conserved among the different HA subtypes [9,61,62,65,66,67], we suggest that the C3LB-HA AVP could inhibit influenza A virus with any HA subtype, as it was found in the antiviral assays with the H1N1 strains and H5N2 strain. 

It is important to point out that both, C1LB-HA and C3LB-HA peptides showed similar antiviral activity against H1N1 and H5N2 strains (Table 1), however their binding sites on the HA and their interactions with linear and discontinuous HA epitopes, were different, even that both AVPs shared a region with the same amino acid residues 279 to 285 (Figure 1D). In contrast the C2LB-HA peptide had a low antiviral activity against the H1N1 strains (Table 1), and the docking analysis against *in silico* predicted linear or discontinuous HA epitopes (Table 2), did not show any interaction with these HA epitopes, suggesting that this interaction could also be important for the antiviral activity, as it was found with the other C1LB-HA and C3LB-HA peptides. 

In summary, even that docking results were limited to HA subtype (H1), they suggested that the antiviral activity by the designed AVPs could be the result of multiple interactions among each AVP and important regions of HA stalk domains, such as the helix A, helix B, loop B, fusion peptide (Figures 7, 8, Table 2). All those interactions could interrupt HA conformational changes required to carry out the membranes fusion event [53,22,30], not allowing the viral genome to be released into the cell. But for that, the peptides have to be bound to the target molecule before the viral particle is internalized by the endocytic vesicle; and also, the interactions between the HA and AVP should be stable at the low endosomal pH [11]. This proposal could be confirmed in the future using new AVPs with specific amino acids changes, or by mutation of specific sites in the HA. However, at the moment it is difficult to find a direct relationship between experimental and docking results. Docking is computational tool which suggests a possible mechanism to explain the antiviral effect of the peptides, due to their interactions with the viral target. But it importantly also, suggests those residues that can be modified or mutated in the future to see how peptide biological properties could be affected. Consequently, we are working on that issue.

## Conclusions

The *in vitro* antiviral assays using AVPs designed through bioinformatics tools showed that this strategy was very useful to design specific peptides targeting important viral glycoproteins. The peptides derived from highly conserved sequences of HA1 and HA2 subunits showed antiviral activity *in vitro* in dose-response manner against two subtypes of influenza virus A with different origins, human, avian, and swine. Docking analysis depicted that these AVP could to be able of interacting with important regions of the HA, thus interfering with its function. This strategy is a potential successful alternative for searching anti-influenza drugs which might be developed into very effective antiviral drugs. But also, this strategy could be applicable for other enveloped viruses. 
